# Economic Evaluation of a Web Application Implemented in Primary Care for the Treatment of Depression in Patients With Type 2 Diabetes Mellitus: Multicenter Randomized Controlled Trial

**DOI:** 10.2196/55483

**Published:** 2024-05-16

**Authors:** Esperanza Varela-Moreno, Maria Teresa Anarte-Ortiz, Francisco Jodar-Sanchez, Azucena Garcia-Palacios, Alicia Monreal-Bartolomé, Margalida Gili, Javier García-Campayo, Fermin Mayoral-Cleries

**Affiliations:** 1 Research and Innovation Unit Costa del Sol University Hospital Marbella Spain; 2 Network for Research on Chronicity, Primary Care, and Health Promotion (RICAPPS) Málaga Spain; 3 Department of Personality, Assessment and Psychological Treatment Faculty of Psychology University of Málaga Málaga Spain; 4 Biomedical Research Institute of Malaga Malaga Spain; 5 Department of Applied Economics Faculty of Economics and Business Administration University of Malaga Malaga Spain; 6 Pharmacoeconomics: Clinical and Economic Evaluation of Medications and Palliative Care Malaga Spain; 7 Network Biomedical Research Center. Physiopathology Obesity and Nutrition (CIBERobn) Carlos III Health Institute Madrid Spain; 8 Department of Clinical and Basic Psychology and Biopsychology Faculty of Health Sciences University Jaume I Castellon Spain; 9 Institute of Health Research of Aragon Zaragoza Spain; 10 Department of Psychology and Sociology University of Zaragoza Zaragoza Spain; 11 Research Network on Preventive Activities and Health Promotion in Primary Health Care (RedIAPP) Madrid Spain; 12 Institut Universitari d'Investigació en Ciències de la Salut University Institute for Research in Health Sciences (IUNICS)- Palma Health Research Institute (IDISPA) University of the Balearic Islands Palma Spain; 13 Mental Health Clinical Management Unit University Regional Hospital of Malaga Malaga Spain

**Keywords:** depression, depressive, type 2, diabetes, diabetic, type 2 diabetes mellitus, eHealth, web-based intervention, efficacy, economic evaluation, cost-effectiveness, cost-utility, randomized controlled trial, RCT, randomized, controlled trial, controlled trials, cost, costs, economic, economics, web based, internet based, CBT, psychotherapy, cognitive behavioral therapy, cognitive behavioral therapy, mental health

## Abstract

**Background:**

Depressive disorder and type 2 diabetes mellitus (T2DM) are prevalent in primary care (PC). Pharmacological treatment, despite controversy, is commonly chosen due to resource limitations and difficulties in accessing face-to-face interventions. Depression significantly impacts various aspects of a person’s life, affecting adherence to medical prescriptions and glycemic control and leading to future complications and increased health care costs. To address these challenges, information and communication technologies (eg, eHealth) have been introduced, showing promise in improving treatment continuity and accessibility. However, while eHealth programs have demonstrated effectiveness in alleviating depressive symptoms, evidence regarding glycemic control remains inconclusive. This randomized controlled trial aimed to test the efficacy of a low-intensity psychological intervention via a web app for mild-moderate depressive symptoms in individuals with T2DM compared with treatment as usual (TAU) in PC.

**Objective:**

This study aimed to analyze the cost-effectiveness and cost-utility of a web-based psychological intervention to treat depressive symptomatology in people with T2DM compared with TAU in a PC setting.

**Methods:**

A multicenter randomized controlled trial was conducted with 49 patients with T2DM, depressive symptoms of moderate severity, and glycosylated hemoglobin (HbA_1c_) of 7.47% in PC settings. Patients were randomized to TAU (n=27) or a web-based psychological treatment group (n=22). This web-based treatment consisted of cognitive behavioral therapy, improvement of diabetes self-care behaviors, and mindfulness. Cost-effectiveness analysis for the improvement of depressive symptomatology was conducted based on reductions in 3, 5, or 50 points on the Patient Health Questionnaire–9 (PHQ-9). The efficacy of diabetes control was estimated based on a 0.5% reduction in HbA_1c_ levels. Follow-up was performed at 3 and 6 months. The cost-utility analysis was performed based on quality-adjusted life years.

**Results:**

Efficacy analysis showed that the web-based treatment program was more effective in improving depressive symptoms than TAU but showed only a slight improvement in HbA_1c_. Incremental cost-effectiveness ratios of 186.76 for a 3-point reduction in PHQ-9 and 206.31 for reductions of 5 and 50 percentage points were obtained. In contrast, the incremental cost-effectiveness ratio for improving HbA_1c_ levels amounted to €1510.90 (€1=US $1.18 in 2018) per participant. The incremental cost-utility ratio resulted in €4119.33 per quality-adjusted life year gained.

**Conclusions:**

The intervention, using web-based modules incorporating cognitive behavioral therapy tools, diabetes self-care promotion, and mindfulness, effectively reduced depressive symptoms and enhanced glycemic control in patients with T2DM. Notably, it demonstrated clinical efficacy and economic efficiency. This supports the idea that eHealth interventions not only benefit patients clinically but also offer cost-effectiveness for health care systems. The study emphasizes the importance of including specific modules to enhance diabetes self-care behaviors in future web-based psychological interventions, emphasizing personalization and adaptation for this population.

**Trial Registration:**

ClinicalTrials.gov NCT03426709; https://clinicaltrials.gov/study/NCT03426709

**International Registered Report Identifier (IRRID):**

RR2-10.1186/S12888-019-2037-3

## Introduction

Depressive disorder and type 2 diabetes mellitus (T2DM) are highly prevalent pathologies, mainly treated in primary care (PC) [1-5]. Their comorbidity has been reported in the previous literature in numerous studies [6-12]. However, the diagnosis of depression in PC is very low and only half of the diagnosed patients receive adequate care [1,2]. Pharmacological treatment remains the alternative of choice despite controversial results [13] and the existence of more effective psychological interventions preferred by patients [14,15]. This situation is due to the lack of available resources [16], the difficulty in accessing face-to-face interventions, and the shortage of professionals [17]. The identification and treatment of depression are essential components in the comprehensive treatment of diabetes since the presence of depressive symptoms in these patients not only affects mood but also has repercussions on all aspects of the person’s life and influences adherence to medical prescriptions [18-20], glycemic control, and the number of future complications [21,22]. All these have an impact on increased health care costs [18,23], so it is necessary to offer comprehensive treatment aimed at improving both psychological and medical outcomes [24].

Information and communication technologies have recently been incorporated into the health care setting (eHealth) as an alternative to improve the continuity and accessibility of treatment, offering economic advantages. In a recent systematic review [25], the authors identified the main randomized controlled trials published to date on eHealth psychological interventions for depression in adults with diabetes.

These programs contributed to improving depressive symptomatology [26-32], but there is still no evidence in improvement in glycemic control. This study aimed to evaluate the efficacy of a low-intensity psychological intervention for the treatment of mild-moderate depressive symptomatology specifically in people with T2DM using a web application compared with treatment as usual (TAU) implemented in PC.

## Methods

### Design

A multicenter randomized controlled trial was conducted. Participants were recruited by family physicians from PC centers in 3 Spanish regions: Andalusia, Aragon, and the Balearic Islands and were randomly assigned to either the intervention group (IG), which included a web-based treatment program, or the control group (CG) that only received TAU.

### Study Procedure

**Figure 1:**
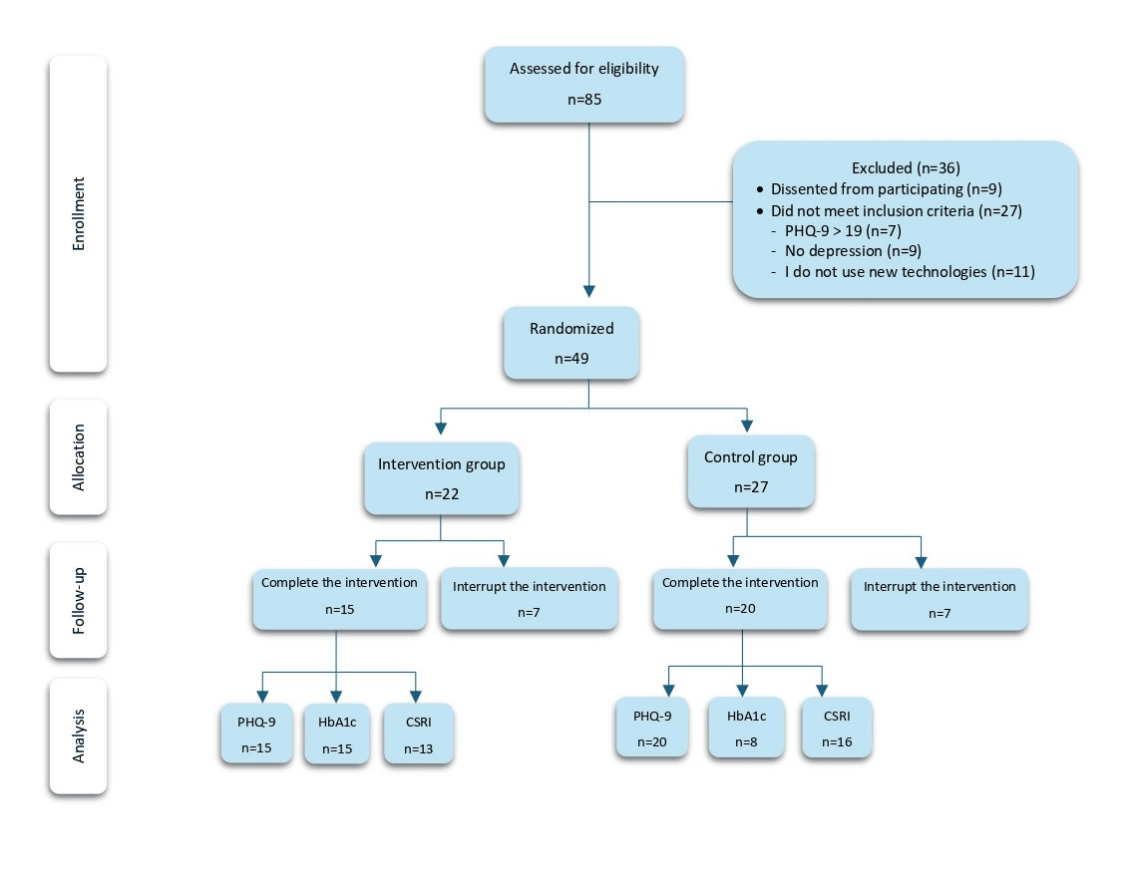
CONSORT (Consolidated Standards of Reporting Trials) flow diagram. CSRI: Client Service Receipt Inventory; HbA1c: glycosylated hemoglobin; PHQ-9: Patient Health Questionnaire–9.

A total of 49 patients with T2DM and depression were recruited. Figure 1 presents the flow diagram of the randomization and patient assessment process from patient selection by PC physicians to 6-month follow-up. Each participant, after being identified by his or her PC physician and signing the informed consent form, was referred to the study investigator for baseline assessment. If they met the criteria, randomization was performed independently by a person outside the study. The inclusion period was from December 1, 2018, to December 31, 2019. Inclusion criteria were adults older than 18 years old, diagnosis of depression (*Diagnostic and Statistical Manual of Mental Disorders fifth edition* [*DMS-5*] criteria) with mild or moderate severity (≤19 points on the Patient Health Questionnaire-9 [PHQ-9]), diagnosis of T2DM (American Diabetes Association), and having an internet connection.

### Ethical Considerations

The research study was approved by the Human Research Ethics Committee of the Regional Health Authority of Aragon (CEICA; PI16/0259) and was designed in accordance with the ethical standards laid down in the Declaration of Helsinki and its later amendments. The research study followed the CONSORT-EHEALTH (Consolidated Standards of Reporting Trials of Electronic and Mobile Health Applications and Online Telehealth) guidelines (Multimedia Appendix 1). All participants signed informed consent forms after being informed of the nature, objectives, potential benefits, and risks associated with participation in the study, as well as the confidentiality of the data collected.

### Interventions

The intervention was based on the cognitive behavioral therapy (CBT) and mindfulness self-help program applied through the internet for the treatment of depression “Smiling is fun” [33], which consists of different therapeutic modules. For this study, in addition to the modules previously designed for “Smiling is fun” [33], a specific module for the promotion of diabetes self-care behaviors, a welcome module, and 2 face-to-face sessions were included. The efficacy of this program has been demonstrated in previous studies [33-36], and the study protocol [37] has been published elsewhere. The CG received only TAU, which refers to treatment administered from PC to patients with depression and T2DM.

### Study Measures

#### The PHQ-9 Questionnaire

Depressive symptomatology was assessed using the PHQ-9 [38], Spanish version [39]. The sum of the responses indicates different levels of depression (0-27). In general, scores above 5 suggest the presence of depressive symptomatology. It presents good internal consistency with a Cronbach α coefficient of 0.89.

#### Glycosylated Hemoglobin

Glycosylated hemoglobin (HbA_1c_) obtained from blood samples was used as an outcome measure of T2DM control, indicating the average blood glucose level during the last 2 or 3 months [40].

#### The 12-Item Short Form Survey Health Questionnaire

To assess the quality of life, the 12-Item Short Form Survey (SF-12) [41], Spanish version [42] of this instrument, was used, which provides a profile of health status and consists of 8 dimensions ranging from 0 (the worst health status for that dimension) to 100 (the best health status). Thus, the higher the score, the higher the perceived quality of life. For this study, the quality-adjusted life years (QALYs) were calculated from the scores of this questionnaire.

#### Consumption of Health Resources and Cost Procedure

The consumption of health services and the social impact were recorded using the Spanish version of the Client Service Receipt Inventory [43] in a self-reported manner by the patient or using the electronic medical record. Data were collected on those resources derived exclusively from depressive symptomatology and T2DM used during the 6 months before and 6 months after the start of the intervention.

### Economic Evaluation

#### Cost Analysis

The economic evaluation was carried out from a social perspective, including indirect and direct health care costs expressed in euros for the year 2018. Direct health care costs were estimated based on the use of health care services according to Andalusian public prices [44] (Table 1). The costs of developing and maintaining the web page were calculated using the equivalent annual cost method. The intervention program included two 25-minute face-to-face sessions with a psychologist, and this cost was estimated according to the minimum interprofessional salary for a psychologist in Spain [45]. The costs of lost productivity (indirect costs) were estimated using the human capital approach, multiplying the minimum interprofessional wage by the number of days of sick leave [46].

**Table 1:** Direct health care costs (financial year 2018; values in €^a^).

Health resources	IG^b^ (€), mean (SD)	CG^c^ (€), mean (SD)
Medical PC^d^	248.08 (364.24)	248.59 (398.31)
Nurse PC	32.29 (82.74)	23.14 (42.73)
Medical PC home	N/A^e^	23.98 (95.92)
Nurse PC home	N/A	5.64 (22.57)
Endocrinology	21.75 (54.26)	21.40 (46.00)
Mental health	30.15 (80.83)	7.13 (28.53)
Emergencies	55.47 (73.04)	27.04 (78.45)
Hospitalizations	N/A	N/A
Total health care cost	387.74 (508.91)	356.94 (500.49)

^a^€1=US $1.18 in 2018.

^b^IG: intervention group.

^c^CG: control group.

^d^PC: primary care.

^e^N/A: not applicable.

#### Efficacy, Cost-Effectiveness, and Cost-Utility

The outcome measures to assess the efficacy of the psychological web-based treatment program were improvements in depressive symptomatology and HbA_1c_. Improvement in depressive symptomatology was estimated based on an improvement in PHQ-9 scores of 3, 5, and 50 percentage points. Efficacy for diabetes control was estimated based on a 0.5% reduction in HbA_1c_. These criteria were determined based on previous studies [28,31,47-51] in the absence of a gold standard criterion.

For each group, cost, incremental cost, efficacy, incremental efficacy, and dominance were calculated, and in the absence of dominance, the results were expressed in terms of incremental cost-effectiveness ratio (ICER).

The unit of measurement for the cost-utility analysis was QALYs, estimated from the SF-12 questionnaire. In addition to QALY, QALY gain was calculated by considering baseline imbalances in utility levels [52]. Similarly, as in the cost-effectiveness analysis (CEA), cost, incremental cost, QALY, incremental QALY, and dominance were calculated for each group, and in the absence of dominance, the results were expressed in terms of incremental cost-utility ratio (ICUR).

The results of the base case were presented in the cost-effectiveness plane that allows us to outline 4 possible scenarios or outcomes after economic evaluation, corresponding to each one of the quadrants of this plan: (1) southeast quadrant—the evaluated technology or new treatment is more effective and less costly than the reference treatment; (2) upper left quadrant—the evaluated technology is less effective and more costly than the reference treatment; (3) southwest quadrant—the evaluated technology is less effective and less costly than the reference treatment; and (4) northeast quadrant—the evaluated technology provides a gain in effectiveness in exchange for equally higher costs.

#### Sensitivity Analysis

A univariate sensitivity analysis was performed to analyze the robustness of the results obtained after cost-effectiveness and cost-utility analysis, assuming a variability of ±20% in the main components of the economic evaluation: health costs, direct costs associated with the intervention, indirect costs, effectiveness of depression (PHQ-9), effectiveness of diabetes (HbA_1c_), and QALYs. These results were presented using a tornado diagram.

### Statistical Analysis

Data collection was performed with Excel (Microsoft Corp), and statistical analysis was performed with SPSS (version 20; IBM Corp), licensed by the University of Malaga. A descriptive analysis of categorical and qualitative variables was performed, using frequencies and proportions. Means and SDs were calculated for quantitative variables. Comparisons between groups were performed using *χ*^2^ tests and the McNemar test for qualitative variables and the 2-tailed Student *t* test or Mann-Whitney *U* test bilaterally for quantitative variables. In all cases, statistical significance corresponded to a P value of <.05.

## Results

### Baseline Characteristics of the Sample

The sociodemographic and clinical characteristics are shown in Table 2; there were no significant differences between the groups before the intervention. At the baseline assessment, moderate depressive symptoms (total score between 10 and 14 on the PHQ-9) were observed in both the IG (mean 12.71, SD 3.60) and the CG (mean 11.81, SD 3.14; P=.37). Regarding the treatment for depressive symptoms (N=49), only 12% (n=6) of the patients had attended mental health consultations, 31% (n=15) of the participants were using antidepressants, and 24% (n=12) of the participants were prescribed anxiolytics. In terms of biomedical variables or diabetes control, the IG had an HbA_1c_ level of 7.16%, while the CG had a baseline HbA_1c_ level of 7.78% (P=.29; P=.51). As for the treatment for diabetes control, 45% (n=22) of the participants were using oral antidiabetic drugs and 12% (n=6) of the participants were using insulin.

**Table 2:** Baseline characteristics of the sample.^a^

Characteristics		Intervention group (n=22)	Control group (n=27)
**Sociodemographic**			
	Age (years), mean (SD)	58.27 (8.01)	56.52 (9.04)
	Female, n (%)	16 (73)	12 (44)
	Married or in a relationship, n (%)	15 (68)	18 (67)
**Educational level, n (%)**			
	No qualification	6 (27)	6 (22)
	High school	12 (54)	11 (41)
	College qualification or more	4 (18)	10 (37)
**Employment status, n (%)**			
	Employed	6 (27)	11 (41)
	Unemployed	7 (32)	10 (37)
	Retired	5 (23)	4 (15)
Depressive symptoms: PHQ-9^b^ (range 5-27), mean (SD)		12.71 (3.60)	11.81 (3.14)
HbA_1c_^c^ (%)^d^		7.16	7.78

^a^Intervention and control groups did not significantly differ (P>.05 in all cases) on any of the sociodemographic or clinical baseline characteristics. Values >5 on the PHQ-9 indicate the presence of depressive symptoms.

^b^PHQ-9: Patient Health Questionnaire-9.

^c^HbA_1c_: glycosylated hemoglobin.

^d^Percentage of glycosylated hemoglobin, absolute values do not apply.

After 6 months from the start of the study, of the total number of patients included, 71% (25/35 participants) of patients with T2DM completed follow-up. The study dropout rate was very similar between both groups, being 36% (8/22) for IG versus 33% (9/27) for CG, with no statistically significant differences. Among the patients in IG (n=22) who dropped out, 14% (n=3) stated that they dropped out because of “lack of time,” 4% (n=1) reported that they dropped out because they did not like the web program, and 18% (n=4) dropped out for “other reasons.” With respect to the number of therapeutic modules completed, 41% (n=9) of the patients completed all modules, 18% (4/22) did not complete any module, and 4% (n=1) completed only 1 module. No statistically significant differences in sociodemographic and clinical characteristics were found between patients who dropped out and those who completed follow-up, as well as between those who completed all modules and those who did not.

### Economic Evaluation

#### Cost Analysis

The mean health care costs associated with PC consultations (medical and nursing consultations in person and at home), endocrinology, mental health, emergency room visits, and hospital admissions were very similar between the 2 groups, being €387.75 (€1=US $1.18 in 2018) in the IG and €356.94 for the CG, with no statistically significant differences between the groups. The development of the web platform had a total cost of €130,123. However, considering that the complete duration of the treatment, including follow-up, is a total of 6 months, the cost of developing the web platform per patient would be €65.06. On the other hand, the implementation of the intervention had associated personnel costs for the development of 2 face-to-face sessions, estimated at €51.10 per patient. The cost associated with productivity losses for the CG or group that received only the TAU from PC was €61.67 per patient. The total costs for the IG were €503.91 per patient and for the CG €418.61 per patient (Table 3).

**Table 3:** Estimated total direct and indirect costs for each study group.

	Intervention group (€^a^), mean (SD)	Control group (€), mean (SD)
Health care costs	387.75 (508.91)	356.94 (500.49)
Web development costs	65.06 (0.00)	N/A^b^
Face-to-face psychological intervention	51.10 (0.00)	N/A
Productivity losses	N/A	61.67 (246.67)
Total costs	503.91 (508.91)	418.61 (567.42)

^a^ €1=US $1.18 in 2018.

^b^N/A: not applicable.

#### CEA as an Outcome Measure for the Improvement of Depressive Symptomatology

**Figure 2:**
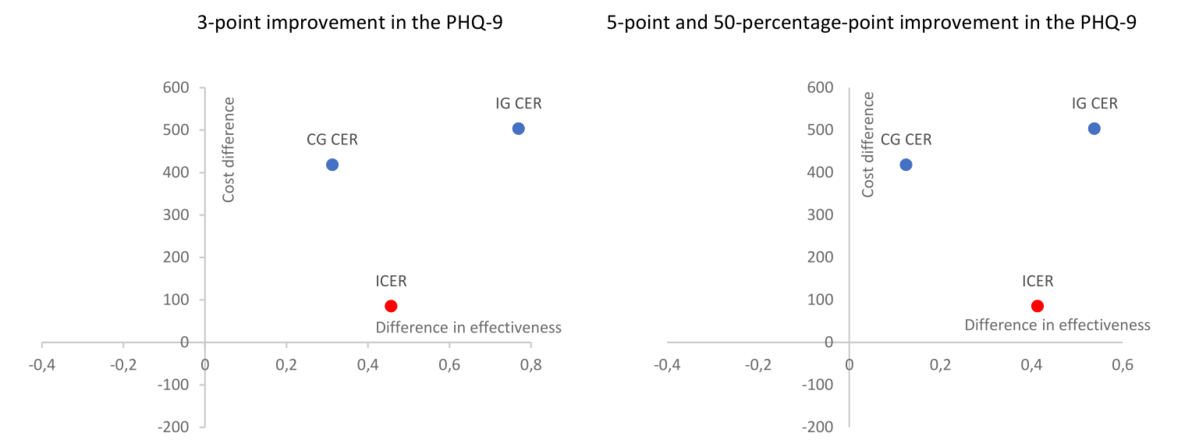
Cost-effectiveness plan for the improvement of depressive symptomatology in patients with type 2 diabetes mellitus after web-based psychological intervention. CG CER: control group cost-effectiveness ratio; ICER: incremental cost-effectiveness ratio; IG CER: intervention group cost-effectiveness ratio; PHQ-9: Patient Health Questionnaire-9.

The web-based psychological intervention proved to be more effective than the TAU in all scenarios analyzed: reduction of 3 points (76.92% vs 31.25%), 5 points, and 50 percentage points in PHQ-9 (53.85% vs 12.50%). The results of the CEA show that this web-based psychological treatment program can be considered an efficient intervention for the control of depressive symptomatology in people with T2DM. In addition, although the IG had a slightly higher mean cost per patient than the CG (€503.91 vs €418.61) mainly due to the costs of the development of the intervention itself, the ICER was €186.76 per patient with no symptoms (for the 3-point scenario) and €206.31 per patient with no symptoms for the 5- and 50-point scenarios (Table 4). In the cost-effectiveness plane, the ICER for the 3 criteria was in the northeast quadrant (upper right), indicating that the web-based intervention was more effective than TAU from PC and at a higher cost (Figure 2).

**Table 4:** Results of the cost-effectiveness analysis of the web-based psychological intervention program to treat depressive symptomatology versus usual treatment in primary care.

		Cost, €^a^, (95% CI)	Efficacy, % (95% CI)	CER^b^ (€)
**3-point reduction in the PHQ-9^c^**				
	Psychological web-based intervention	503.91 **(**266.65 to 794.84)	76.92 **(**54.00 to 99.80)	655.08
	TAU^d^	418.61 **(**179.75 to 725.94)	31.25 **(**8.50 to 54.00)	1339.55
	Incremental	85.30 **(**–325.51 to 496.11)	45.67 **(**13.40 to 77.90)	186.76
**5-point reduction in the PHQ-9**				
	Psychological web-based intervention	503.91 **(**266.65 to 794.84)	53.85 **(**26.70 to 80.90)	935.83
	TAU	418.61 **(**179.75 to 725.94)	12.50 **(**–3.70 to 28.70)	3348.87
	Incremental	85.30 **(**–325.51 to 496.11)	41.35 **(**9.80 to 72.90)	206.31
**50 percentage point reduction PHQ-9**				
	Psychological web-based intervention	503.91 **(**266.65 to 794.84 )	53.85 **(**26.70 to 80.90)	935.83
	TAU	418.61 **(**179.75 to 725.94)	12.50 **(**–3.70 to 28.70)	3348.87
	Incremental	85.30 **(**–325.51 to 496.11)	41.35 **(**9.80 to 72.90)	206.31
**0.5 reduction in HbA_1c_^e^**				
	Psychological web-based intervention	481.59 **(**231.05 to 801.47)	8.33 **(**–7.30 to 24.00)	5779.13
	TAU	571.53 **(**179.44 to 1177.61)	14.29 **(**–11.60 to 40.20)	4000.70
	Incremental	–89.93 **(**–773.18 to 593.31)	–5.95 **(**–36.20 to 24.30)	1510.90

^a^ €1=US $1.18 in 2018.

^b^CER: cost-effectiveness ratio.

^c^PHQ-9: Patient Health Questionnaire-9.

^d^TAU: treatment as usual.

^e^HbA_1c_: glycosylated hemoglobin.

#### CEA as an Outcome Measure of Improvement in HbA1c Levels

**Figure 3:**
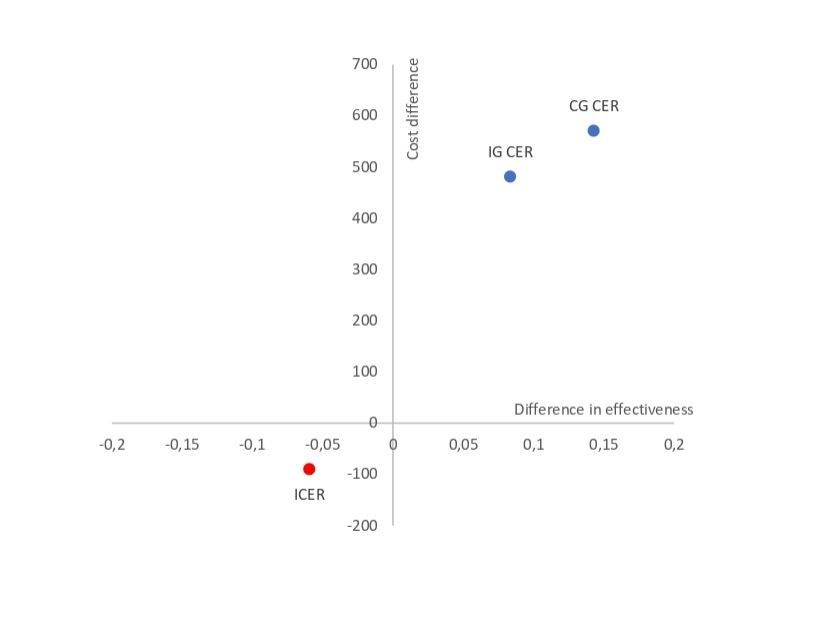
Cost-effectiveness plan for the improvement of HbA1c control in patients with type 2 diabetes mellitus after web-based psychological intervention. CG CER: control group cost-effectiveness ratio; ICER: incremental cost-effectiveness ratio; IG CER: intervention group cost-effectiveness ratio.

The web-based psychological intervention to improve HbA_1c_ levels in people with T2DM is reduced in IG compared with CG (8.33% vs 14.29%). Furthermore, although IG had a slightly lower cost per patient than CG (€481.59 vs €571.53), the ICER was €1510.90 (Table 4). In the cost-effectiveness plane, the ICER is in the southwest quadrant (lower left), indicating that the web-based intervention was less effective and less costly than the TAU from PC (Figure 3).

#### Cost-Utility Analysis

**Figure 4:**
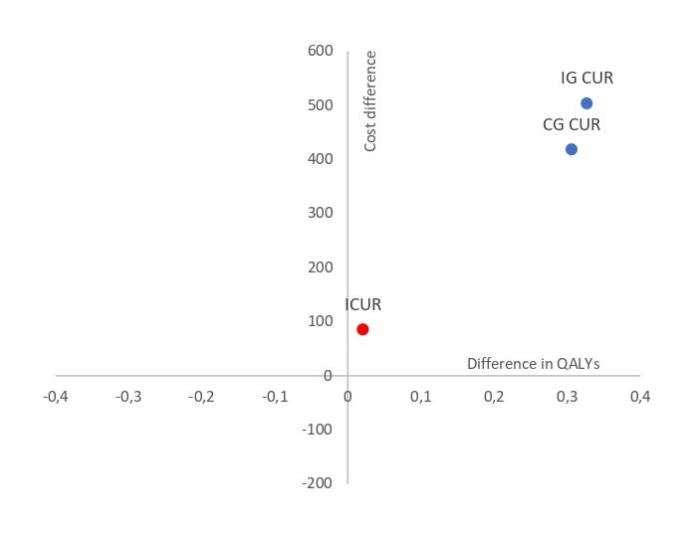
Cost-utility per QALY of the web-based psychological intervention program for the improvement of depressive symptomatology in people with type 2 diabetes mellitus. CG CUR: control group cost-utility ratio; ICUR: incremental cost-utility ratio; IG CUR: intervention group cost-utility ratio; QALYs: quality-adjusted life years.

Both study groups started from a baseline level of utility that was practically similar (QALY 0.5898 vs 0.5935). The ICUR was €4119.33 per QALY. Therefore, the ICUR shows that the group that received the web-based psychological intervention program from PC achieved greater health gains (incremental QALY of 0.0207) and a higher cost compared to the group that only received TAU. Regarding the effect on QALY gained, this was higher in the IG (0.0321) than in the CG (0.0095). The ICUR resulted in 4174.92 per QALY gained. Therefore, the CUA shows that the group receiving the web-based psychological intervention program from PC achieved greater health gains (incremental QALY gain of 0.0225) and a higher cost compared to the group receiving only TAU (Table 5). Figure 4 shows the results of the cost-utility plane using QALY and QALY gained as the benefit outcome measure. ICUR is in the northwest quadrant (lower left), indicating that the web-based psychological intervention is more costly but contributes to increased QALYs.

**Table 5:** Results of the cost-utility analysis of the web-based psychological intervention program for the improvement of depressive symptomatology versus usual treatment.

			Cost, €^a^ (95% CI		Gain (95% CI)		CUR^b^, €
**QALYs^c^**							
	Psychological web-based intervention	503.91 (266.65 to 794.84		0.3270 (0.2920 to 0.3623		1541.10	
	TAU^d^	418.61 (179.75 to 725.94		0.3063 (0.2840 to 0.3278		1366.78	
	Incremental	85.30 (–325.51 to 496.11		0.0207 (–0.0229 to 0.0643		4119.33	
**QALYs gained**							
	Psychological web-based intervention	503.91 (266.65 to 794.84		0.0321 (0.0133 to 0.0512		15718.80	
	TAU	418.61 (179.75 to 725.94		0.0095 (–0.0036 to 0.0234		43955.57	
	Incremental	85.30 (–325.51 to 496.11		0.0225 (–0.0023 to 0.0474		3785.36	

^a^ €1=US $1.18 in 2018.

^b^CUR: cost-utility ratio.

^c^QALYs: quality-adjusted life year.

^d^TAU: treatment as usual.

#### Sensitivity Analysis

**Figure 5:**
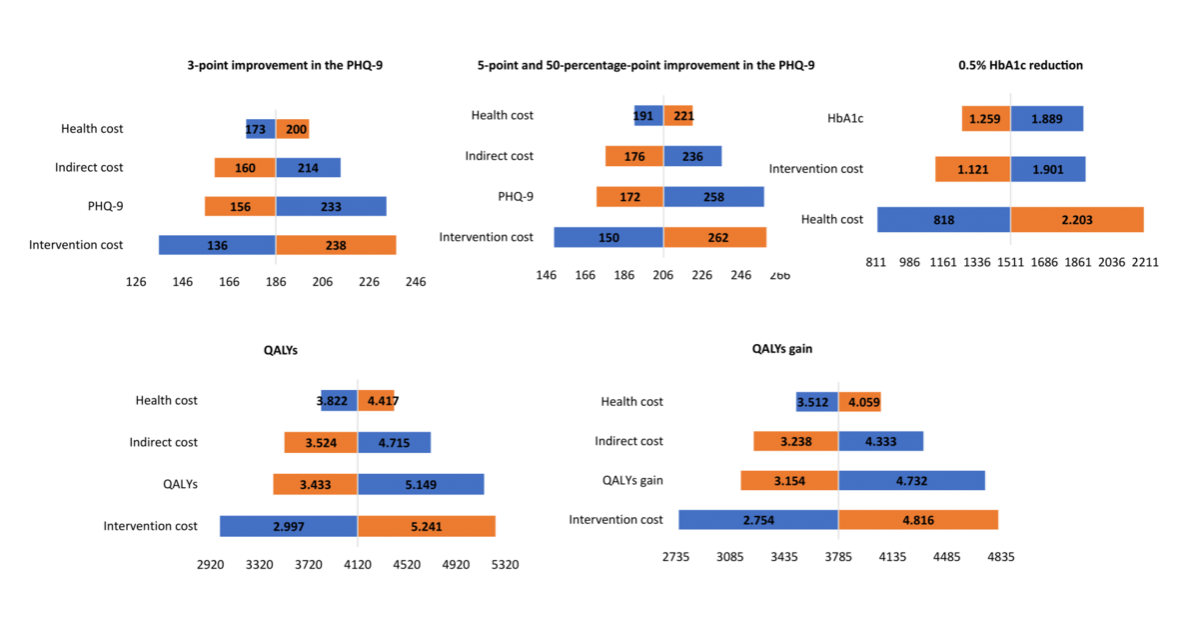
Sensitivity analysis for a web-based psychological intervention program. HbA1c: glycosylated hemoglobin; PHQ-9: Patient Health Questionnaire-9; QALYs: quality-adjusted life years.

Sensitivity analysis again showed that the ICER was cost-effective and that the results obtained were robust with respect to the base case. The variable that generated the most uncertainty in almost all cases was the costs of the intervention. However, taking a reduction in HbA_1c_ as a reference, the variable that generated the most uncertainty was health care costs. These results can be seen in Figure 5. Taking as a reference the results obtained based on a reduction in the PHQ-9 as a criterion for improving depressive symptomatology, the variable that generated the most uncertainty was the costs of the intervention, as shown in Figure 5. The results were obtained based on a reduction of 3 points on the PHQ-9 as a criterion for improving depressive symptomatology, and the variable that generated the most uncertainty was the costs of the intervention, with oscillations of between €136 per patient with no depressive symptomatology and €238 per patient with no depressive symptomatology, considering the best- and worst-case scenarios. In criterion B and C, reduction of 5 points and 50 percentage points on the PHQ-9. The variable that generated the most uncertainty was also the cost of the intervention, with oscillations of between €150 per patient without depressive symptomatology and €262 per patient without depressive symptomatology.

The results of the sensitivity analysis for the ICUR, using QALYs as a measure of benefit, were also robust to the base case. Figure 5 shows how variations are associated with oscillations between €2997 per QALY and €5241 per QALY for patients with no depressive symptomatology. On the other hand, variations in health care costs ranged from €3822 per QALY to €4417 per QALY for patients with no depressive symptomatology. The results of this analysis for the ICUR using the QALY gain as a measure of benefit obtained results are similar to those described in the case of QALYs. These results show that they are cost-utility (Figure 5).

## Discussion

### Principal Findings

This study performed the economic evaluation of a psychological intervention based on a web-based treatment with CBT and mindfulness to reduce depressive symptomatology and improve diabetes control. Although this type of intervention has been analyzed in other studies [26,28,30,31,53-56], this is the first study developed specifically for people with T2DM, not including patients with other types of diabetes (eg, patients with type 1 diabetes mellitus together with patients with T2DM) who, therefore, have different treatments and characteristics. It also includes a specific module aimed at promoting diabetes self-care behaviors and implemented directly by the Spanish PC health system.

The results of the CEA show that the intervention proved to be more effective and more costly in reducing the symptoms of depression compared to the TAU group. These results are to be expected after analyzing novel interventions developed using information and communication technologies and are in line with previous studies designed for the general population [36,57-61]. For a population with diabetes, only the study presented by Nobis et al [62] published results of CEA and CUA of a similar intervention, obtaining results that are equally in line with those obtained by this study.

Finally, the CUA results show that the intervention improved quality of life, measured in terms of QALYs, compared with TAU in PC. These results, with ICUR of €4119 per QALY and €3785 per QALY gained, are below the threshold value of between €22,000 and €25,000 per QALY estimated in Spain [63,64] as the maximum willingness to pay per QALY gained. Compared with previous studies, our results are very promising. Warmerdam et al [65] found that the additional cost of obtaining a QALY after the use of web-based CBT was €22,609 compared with the waiting list group, which is much higher than the results obtained in this study. Nobis et al [62] concluded that treatment response was not reflected in QALY gains, and the extra cost of obtaining a QALY amounted to €14,000. Romero-Sanchiz et al [36] have been the only economic analysis study on a web-based psychological intervention performed so far in Spain in the PC setting in depression and showed a reduction in the cost of €11,390.

This study has limitations. First, difficulties were encountered in obtaining blood samples and determining HbA_1c_ during the follow-up phase. These difficulties were generated by the COVID-19 confinement. However, despite this circumstance, the results obtained are similar to those reported in previous studies [28-31,53,54] on face-to-face psychological interventions. Second, the cost analysis did not include the consumption associated with drugs for depression or diabetes. However, the reported medication costs were equally distributed between the 2 groups, so they would not make much difference to the results of the economic evaluation [36]. Finally, there were limitations in defining the criteria for the efficacy of the intervention because there was no gold standard for improvements in depressive symptomatology or HbA_1c_.

Although the results are promising, more studies are needed because the evidence on the cost-effectiveness of these interventions is scarce. Therefore, studies are needed to replicate this intervention, increasing the sample size to be able to compare and generalize the results obtained. Likewise, the current challenge is directed toward the design of web-based psychological interventions in a personalized way according to the pathology to be treated to achieve better results and better adherence to them [66,67].

### Conclusions

This intervention, devised to reduce depressive symptoms and enhance glycemic control among individuals with T2DM through a series of online web-based modules incorporating CBT tools, promotion of diabetes self-care behaviors, and mindfulness, has proven to be both effective and efficient. This effectiveness extends to both clinical outcomes and economic considerations. Consequently, these eHealth interventions not only facilitate clinical improvements for patients but also demonstrate cost-effectiveness for health care systems.

The findings of this study underscore the significance of incorporating a specific module focused on enhancing diabetes self-care behaviors. This element is deemed indispensable for the future development of web-based psychological interventions, ensuring their personalization and adaptation to the unique needs of individuals within this diabetic population. Moreover, the study supports the notion that interventions targeting the improvement of depressive symptoms in individuals with diabetes, while essential, may not be adequate in isolation for optimizing the overall clinical management of diabetes [25,30,68-71].

In summary, this intervention not only directly benefits patients by improving their mental and physical health but also brings efficiency and adaptability to health care settings, highlighting its importance in the effective management of diabetes in diverse health care settings.

## Appendix

Multimedia Appendix 1 CONSORT-EHEALTH (Consolidated Standards of Reporting Trials of Electronic and Mobile Health Applications and Online Telehealth) checklist.

## Abbreviations

CBT: cognitive behavioral therapy
CEA: cost-effectiveness analysis
CG: control group
CONSORT-EHEALTH: Consolidated Standards of Reporting Trials of Electronic and Mobile Health Applications and Online Telehealth
CUA: cost-utility analysis
DSM-5: Diagnostic and Statistical Manual of Mental Disorders, fifth edition
HbA1c: glycosylated hemoglobin
ICER: incremental cost-effectiveness ratio
ICUR: incremental cost-utility ratio
IG: intervention group
PC: primary care
PHQ-9: Patient Health Questionnaire-9
QALY: quality-adjusted life year
SF-12: 12-Item Short Form Survey
TAU: treatment as usual
T2DM: type 2 diabetes mellitus
